# Modern drug design: the implication of using artificial neuronal networks and multiple molecular dynamic simulations

**DOI:** 10.1007/s10822-017-0085-7

**Published:** 2017-11-13

**Authors:** Oleksandr Yakovenko, Steven J. M. Jones

**Affiliations:** 1 Ifowonco Inc, Vancouver, Canada; 2 Genome Sciences Centre, BC Cancer Agency, Vancouver, Canada

**Keywords:** Molecular dynamic, Machine learning, Computational drug design, D3R, Drug Design Data Resource

## Abstract

We report the implementation of molecular modeling approaches developed as a part of the 2016 Grand Challenge 2, the blinded competition of computer aided drug design technologies held by the D3R Drug Design Data Resource (https://drugdesigndata.org/). The challenge was focused on the ligands of the farnesoid X receptor (FXR), a highly flexible nuclear receptor of the cholesterol derivative chenodeoxycholic acid. FXR is considered an important therapeutic target for metabolic, inflammatory, bowel and obesity related diseases (Expert Opin Drug Metab Toxicol 4:523-532, 2015), but in the context of this competition it is also interesting due to the significant ligand-induced conformational changes displayed by the protein. To deal with these conformational changes we employed multiple simulations of molecular dynamics (MD). Our MD-based protocols were top-ranked in estimating the free energy of binding of the ligands and FXR protein. Our approach was ranked second in the prediction of the binding poses where we also combined MD with molecular docking and artificial neural networks. Our approach showed mediocre results for high-throughput scoring of interactions.

## Introduction

The 2016 drug design Grand Challenge 2 competition consisted of six blind sub-challenges focused on the FXR protein: (a) the ligand-based prediction of binding potency for 102 compounds with an experimentally measured affinity, (b) the prediction of spatial coordinates of 36 molecular complexes with experimentally resolved atomic coordinates (pose prediction), (c) the high-throughput prediction of binding potency by structure based methods (before and after disclosure of the 36 experimentally resolved complexes) for the 102 compounds and (d) relative free energy estimations within the subset of homologous compounds (two subsets of 15 sulfonamides and 18 spiros compounds). We participated in four sub-challenges: we were ranked first and second respectively in the free energy and pose predictions sub-challenges. Our approach showed mediocre accuracy in the high-throughput structure-based virtual screening sub-challenges both before and after the release of the 36 experimentally resolved binding poses.

The goal of structure-based high throughput virtual screening is to find and evaluate energetically favorable binding modes between a target protein and millions of candidate small organic compounds in a timeframe for which one is willing to tolerate. Current approaches for the scanning of possible binding configurations usually exclude the majority of the protein’s internal structural degrees of freedom with the goal of only sampling a sufficient number of conformations for a small flexible compound. While it is believed that docking algorithms perform reasonably well in determining the geometries of the docked complexes, they usually fail to accurately evaluate the corresponding free energy of binding [2, 3]. This may not be surprising since even an approximate estimation of entropy—the major term describing free energy in biomolecular complexes—requires sampling over at least the typically observed range of internal conformational degrees of freedom of all the participating molecules. Simulations of the molecular dynamics are considered the most established and physically robust methods for routinely scanning through the probable states of the molecular ensembles. MD, in principle, provides a good estimation of the entropy contribution to the binding free energy of a compound and is usually carried out through a cohort of specialized techniques, such as free energy perturbation, thermodynamic integration, umbrella sampling, Jarzynski non-equilibrium pulling, multiple replica scaled regression models, explicit multiple routes sampling (from long MD) and even simplified linear interaction energy (LIE) methods [4–9]. Unfortunately, the MD approach can overwhelm available computational resources, which makes it impractical for all but the smallest throughput virtual screening experiments. As a potential solution, we are developing a machine learning (ML) approach, inspired by advances in machine vision algorithms, that can effectively generalize practical rules by observing only a limited subset of robust physical MD simulations, and then use the learned experience to examine and classify a large set of binding predictions generated by a much less computationally demanding molecular docking experiment.

**Fig. 1:**
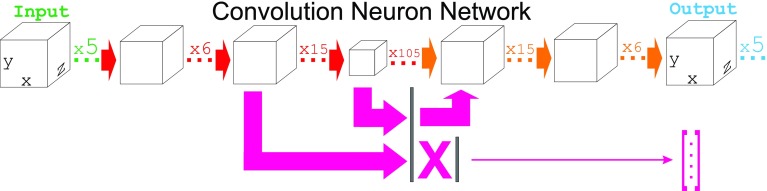
ANN architectures trained to predict a MD outcome from docking poses. The 3D (de-)convolutional layers are shown with boxes and perceptron layers are shown with flat vertical lines. The data flow (from left to right) is shown with arrows: red—convolutional encoder, orange—convolutional decoder, magenta—energy predicting perceptron. The ‘X’ represents fully connected layers within the model. The NN output is a voxel image and a vector of predicted LIE features

Artificial neural networks (ANN), including convolutional neural networks (CNN), are machine learning models that can possess a large learning capacity [10]. CNNs have limited connectivity between neurons of consecutive layers and therefore possess a smaller amount of trainable parameters but typically display only a modest decrease in performance relative to fully connected ANNs. CNNs can be a relevant choice in cases where several first layers of local patterns in input signals can be helpful in generalizing them into more abstract categories. Important examples of such problems are in machine vision and speech recognition where the CNN fragment can generalize pixels into lines and ellipses or sounds into words. Convolution layers are also useful for generalization of proximal patterns in molecular complexes because all the bonded interactions are local at an angstrom scale and non-bonded van-der-Waals interactions decrease as rapidly as ~ r^−6^ with only electrostatic interactions being distant. In practice, a combination of convolutional and fully connected layers is usually used in machine vision or speech recognition so that the convolution layers work until signal proximity is important and then fully connected layers can perform a classification using their more generalized representation [11]. In this work, we generally follow a similar strategy with the difference that our fully connected layers are located within the middle of the convolution encoder-decoder chain (see Fig. 1). The rationale of this design is to introduce predictive perturbations into the encoder-decoder data flow as our aim is to synthesize the evolved geometries instead of the input ones. For predictive perturbations we define an additional interconnection between the convolution encoder and the LIE prediction preceptor which in our model couples otherwise independent hypothesis generating channels.

Deep learning systems, as CNN implementations are often referred to, have been previously used to create a function that predicts the free energy of molecular binding (a score) using the structural information generated by docking software [12]. Our approach is inspired by that approach but it principally differs in how the deep learning generalization is formulated. Particularly, in this study we are not estimating free energy or score but constructing a neural network approach which is able to learn the complex relationships between the complexed molecules and their behavior over time under conditions of a NPT (constant pressure) thermodynamic ensemble. In our opinion, an artificial intelligence (AI) approach for drug design can be built upon a ANN that is focused on predicting fundamental thermodynamic quantities (like receptor conformational entropy) and trained on results of rigorous computational modeling such as MD. As a consequence, such machine learning solutions will be capable of working with very limited sets of experimentally known binders, relying, instead, on deep understanding of the fundamental physical principles of their interactions.

A recent Schrodinger Inc. (USA) implementation of the automated free energy perturbation protocol FEP+ (https://www.schrodinger.com/fep) had biased field of free energy calculations toward an alchemical ligand transmutation approach. It is difficult to overestimate the contribution of Schrodinger Inc to practical drug modeling as they have turned free energy calculations into the de facto standard metric for industrial drug design. We maintain, however, that pulling techniques that actively assess the drug-ligand interaction have the potential to provide considerably higher accuracy in practice. The rationale behind our speculation is that alchemical and pulling approaches solve the biggest problem in FEP modeling—finding and sampling the lowest energy transformation routes on potential energy surface (PES)—with very different strategies. While alchemical FEP+ and similar methods described in the literature [13, 14] seek low-energy routes via direct sampling in the space of chemical compounds and indirectly scanning the space of receptor conformations, pulling methods [15–22] are focused on the direct exploration of protein conformation space. Although both approaches are formally correct, in our opinion, the pulling methods are in a better position to derive accurate estimations, because the sampling of protein conformation space, although being considerably more difficult, describes the interaction more fully than sampling in space small organic compounds. We propose a further modification of the pulling approach, which is specially designed to determine more accurate estimations of binding potency by involving multiple dissociation routes in sampling process. A good illustration of the multiple routes description of the dissociation process, using purine nucleoside phosphorylase as an example, is provided by Decherchi et al. [9]. The advantages of our free energy estimation method are best illustrated by the explicit involvement of so-called orthogonal coordinates in the description of system transition along reaction coordinates on the PES. The orthogonal coordinates are other, independent from the reaction coordinate, degrees of freedom, which strongly affect the shape of the PES. As an example of these orthogonal coordinates we can consider a dihedral angle that triggers the “open” or “closed” state of an allosteric protein loop—although the angle value itself does not define bound and unbound states of a compound, the value strongly correlates with the height of hills on the PES over which an interacting compound dissociates. In such model, the potential of mean force (PMF) is a sum of integrals over PES, which are weighted by the probability density of values of corresponding orthogonal coordinates. Consecutively, the most accurate approach to estimate PMF is to sample both—orthogonal and reaction coordinates. Unfortunately, proteins are very complex and, generally, there is no constructive approach to define the important orthogonal coordinates for a given ligand-receptor pair. To attempt to address this uncertainty, our free energy method combines ideas from Jarzynski non-equilibrium pulling [23] and quasi-equilibrium umbrella sampling [24].

Jarzynski non-equilibrium pulling method computes PMF estimations by repeatedly measuring the work spent on molecular separation in a non-equilibrium artificial pulling process. The equilibrium PMF is estimated as average of many (typically several hundred) non-equilibrium pulling replications. The pulling experiments themselves have to be very short, typically several nanoseconds or even less, in order to complete an entire batch within a reasonable computational budget. With such a short simulation time, the system will be unlikely to relax as the reaction coordinate proceeds, and its trajectory will be close to a straight line in the space of generalized orthogonal coordinates. Thus, this method is essentially a route sampling approach with the main drawback being the poor sampling of low-energy processes. For the routes sampling described within this paper we consider finding (and evaluating) only representative set of sufficiently distinct smooth low-energy dissociation trajectories. By contrast, umbrella sampling is a quasi-equilibrium method with perfect relaxation sampling and typical simulation duration of tens, or hundreds even, of nanoseconds per window. Thus, the umbrella sampling method draws a line that perfectly curls along low-energy pathways in the space of generalized orthogonal coordinates. But, to remain within a reasonable computational budget, only one route is typically sampled and the major error of the method comes from this insufficient sampling of the different routes. We thus combine Jarzynski non-equilibrium pulling and umbrella sampling into a hybrid method whereby reasonable routes of sampling and individual trajectory curling are constructed within an affordable computational budget. Our approach was prototyped and described previously [25] and here we have further extended it with an formal assessment of PMF convergence for the tracking of the estimation outliers.

## Materials and methods

In all our protocols described in this section, molecular dynamic simulations are combined with variety of other methods. In particular, (i) for the pose predictions sub-challenge we used a combination of MD with molecular docking and artificial neural network implementations; (ii) for large-scale compound scoring we used replicated LIE MD runs of the complex in the bound state and (iii) for free energy estimations we used parallel non-equilibrium MD pullings followed by quasi-equilibrium umbrella samplings and assessed PMF convergence as an ad-hoc procedure for the detection of outliers. Although in the Grand Challenge competition we used only four parallel pullings and umbrella samplings, we believe that with more computation resources our hybrid free energy estimation method can be even further extended toward the usage of Jarzynski inequality for an even more accurate PMF evaluation.

### 4D molecular docking

4D Docking, also known as ensemble docking, is a protocol for pose generation, which independently repeats the docking of each compound into each receptor model to from a set of preselected structures. To construct binding poses we used the Autodock Vina docking software [26]. Three known FXR conformations (PDB accession codes 3DCT, 3OOF and 3BEJ) were manually selected for 4D docking and up to 20 top-scored poses for each conformation (up to 60 poses per compound in total) were aggregated and analyzed. The choice of the three crystal structures was motivated by the authors previous drug design experience and the rationale to provide a small but representative set of distinct protein conformations. As such, the selected poses are not guaranteed to be the optimal ones and only reflect author’s understanding of FXR docking problem. Throughout this manuscript we refer to the structure generated with 4D docking (or reported in literature) as the “structure after docking” to distinguish them from structures generated through MD simulation.

### MD simulations

MD simulations were carried out with the GROMACS-4.5.5 MD package [27]. For all complexes, the force field parameters were added using our heuristic: for the protein part AMBER-ILDN parameters [28] as implemented in GROMACS, for compounds bonded parameters from GAFF [29] were used together with Kirchhoff Coulomb charges [30] and Leonard-Jones parameters from YFF1 [31] which were additionally fit for better correspondence with the AMBER-ILDN parameters. The fit was scaled using Leonard-Jones interaction constants with two constant multipliers (one for repulsive and one attractive components) computed by the least square fit of interaction potentials for all matching atom types in YFF1 and AMBER-ILDN.

All MD simulations were carried out with Leonard-Jones and Coulomb short-range interactions cut off at 1.4 and 0.9 nm respectively and neighbor-searching updates made for each ten steps of the MD integrator. Long-range electrostatic interactions were modeled with the particle mesh Ewald algorithm [32]. To model solvated complexes, the structures were placed in periodic box with TIP3 explicit water molecules and 0.1 M of NaCl and then relaxed by l-bfgs minimization followed by 50 ps of MD under a constant volume (NVT) ensemble with restrained positions of protein and the compound heavy atoms. Simulated annealing [33] was used to warm up the system from the initial velocities assigned accordingly to the Boltzmann distribution at the temperature of boiling nitrogen T = 77 K to body temperature T = 310 K. Following this NVT warm up, 100 ps of constant pressure (NPT) equilibration was performed. Complex and other atoms were coupled to a separate temperature coupling baths and the temperature was maintained at T = 310 K. For equilibration, the weak coupling [34] was used to maintain pressure isotropically at 1.0 bar and the Berendsen weak coupling method was sued to maintain constant temperature. All subsequent productive runs were performed with the more accurate Nose–Hoover thermostat [35, 36] with a temperature coupling time constant of 0.1 ps and the Parrinello-Rahman barostat [37] with a pressure coupling time constant of 1.0 ps under a NPT ensemble. This combination of thermostat and barostat ensured that a true NPT ensemble was sampled.

For LIE analyses, we carried out 8 MD simulations with a random seed for Maxwell velocities generations for 16.2 ns each for every analyzed pose: 0.2 ns warmup, 12 ns of complex relaxation and 4 ns of statistic collection. Complex coordinates, together with Leonard-Jones and Coulomb interaction energies of the binding site residues and compound, were collected every 0.05 ns. For the binding site we manually defined a list of FXR residues {265, 270, 273, 284, 286, 287, 288, 290, 291, 294, 325, 328, 329, 331, 332, 335, 336, 352, 357, 365, 369, 384, 447, 451, 454, 461, 465, 469}. Because our free energy estimations using an artificial intelligence approach had not yet been implemented by the time of the competition, we used the LIE free energy equation to rank poses. LIE free energy estimations were calculated accordingly to:

1 $$\begin{array}{*{20}{c}} {\Delta {G_i}=\alpha \Delta {{\left\langle {U_{{LIG}}^{{Coul}}} \right\rangle }_i}+\beta {{\left\langle {U_{{LIG}}^{{LJ}}} \right\rangle }_i}+\gamma {{\left\langle {U_{{site}}^{{Coul}}} \right\rangle }_i}+\delta {{\left\langle {U_{{site}}^{{LJ}}} \right\rangle }_i}+\varepsilon {S_i}} \\ {{w_i}=\frac{{\Delta {G_i}}}{{k\sum {\Delta G} }}} \\ {\Delta {G_{LIE}}=\alpha \sum {{w_i}\Delta {{\left\langle {U_{{LIG}}^{{Coul}}} \right\rangle }_i}} +\beta \sum {{w_i}{{\left\langle {U_{{LIG}}^{{LJ}}} \right\rangle }_i}} +\gamma \sum {{w_i}{{\left\langle {U_{{site}}^{{Coul}}} \right\rangle }_i}} +\delta \sum {{w_i}{{\left\langle {U_{{site}}^{{LJ}}} \right\rangle }_i}} +\varepsilon \sum {{w_i}{S_i}} } \end{array}$$

where *S* is the loss of rotational and translational entropy of the compound (computed according to a quasi-harmonic approximation), α, β, γ, δ and ε are fitting parameters and *k* = 0.2 in our protocol. To determine the training set for the LIE parameters we used seven experimentally resolved structures (PDB accession codes 3BEJ, 3FLI, 3OKH, 3OKI, 3OOF, 3OOK, 3RUT) and five manually constructed complexes with other close homologues compounds reported in literature [38, 39]. The fitted parameters set was α = – 0.059274, β = 0.343518, γ = 0.012304, δ = 0.110224, ε = − 0.00003, U_0_^Coul^ = + 6418.28 kJ mol^− 1^ U_0_^LJ^ = – 1458.75 kJ mol^− 1^.

To generate crystal-like complexes after the simulations, an additional cooling down run was performed for 0.2 ns. For this run, the geometry (and velocities) were taken from the last frame of productive MD simulation and then proceeded using the thermostat as described above but where the temperature was linearly decreased in time until reaching the boiling point of liquid nitrogen by the end of the simulation. Due to the final low temperature, the protein-drug complexes are frozen into a single structure after the MD simulations. Such behavior approximately corresponds to the typical conditions of X-ray experiments where freezing liquid nitrogen is also used. Through this manuscript we refer to the frozen structures after a MD simulation cool down run as the “structure after MD” to distinguish them from structures generated by molecular docking or X-ray refinement.

Four independent dissociation trajectories per compound were generated from the same starting structure of its complex with the FXR protein by applying a forced pulling for 3.2 ns for each at a slightly different but physiological temperatures of 300, 305, 310 and 315 K (one run at each temperature). In all cases the artificial spring force constant was 1000 kJ mol^−1^ nm^−2^ and the pull rate was 1.0 nm ns^−1^. FXR coordinates were manually aligned to superimpose the active site entrance with Z coordinate axis, compounds were set as mobile fragments and the binding site of FXR as a reference group, i.e. a group where the center of mass is used as the reference point for computing the distance to the mobile fragment (compound). The reference group was not constrained but the coordinates of the atoms in the anchoring fragment (Cα atoms of residues 379Q-391L) were restrained with restraining potential 1000 kJ mol^−1^ nm^− 2^ and the pulling was performed along z-axis (0., 0., + 1.). The four pulled trajectories were sampled with umbrella sampling technique under 22 windows centered at the reaction coordinate ξ = {0.0, 0.1, 0.2, 0.3, 0.4, 0.5, 0.6, 0.7, 0.8, 0.9, 1.0, 1.2, 1.4, 1.6, 1.8 2.0, 2.2, 2.4, 2.6, 2.8, 3.0, 3.2} nm of artificial separation relative to equilibrium complex geometry and for a duration of 32 ns per window.

The standard free energy of binding, Δ*G* ^*o*^, was computed from multiple independent umbrella PMFs, *W*, in two stages. At the initial step, the results of the individual runs were averaged (with an arithmetic mean):

2 $$\Delta W=\sum\limits_{i} {{p_i}} \Delta {W_i} \approx \overline {{\Delta W}}$$

where *p* is the probability of a dissociation process following a scenario which is close (in the sense of proximity, which can be covered by finite time umbrella sampling) to the one described by *i*-th trajectory and *W*_*i*_ is estimation of work which is required to dissociate along the *i*-th trajectory. Due to small amount of runs we used an arithmetical average in (2) instead of the well-known Jarzynski inequality $\exp \left( { - {{\Delta W}}/{{RT}}} \right) \leq \overline {{\exp \left( { - {{\Delta {W_i}}}/{{RT}}} \right)}}$. Although Jarzynski inequality is physically correct (and recommended for use with routes coverage of c.a. 16 independent trajectories), the arithmetical average is better for the small number (four in this case) of parallel umbrella runs we conducted. The rationale behind the arithmetic average is that if several distinct but well smoothed trajectories are suggested to form a reasonable representation of the most probable dissociation scenarios (which is a good approximation for many practical cases), then probability of system to dissociate along each of them is also expected to be similar (otherwise it contradicts the assumption about a sufficient equilibration). Therefore, any large differences in umbrella sampling estimations are interpreted as errors and the simple average is preferred over exponential averaging to negate the outliers. In this particular setup, four independent dissociation trajectories starting from the same complex structure were generated at a variety of physiological temperatures as described above and sampled at 310 K. Then, the averaged PMF was converted into a standard free energy of binding. Following [40] we determine the standard free energy of binding as the sum of two terms—PMF work, normalized to the meaning of the bound state (an integral in denominator) and a normalization of the actually sampled unbound volume, *V*_*unbound*_, to the volume of a standard concentration of 1M *V* ^*o*^ = 1661 A^3^:

3 $$\Delta {G^0}=\Delta {G_{PMF}}+\Delta {G_V}= - RT\,\ln \left[ {\frac{{\int {\left( {\exp \left( { - \frac{{W\left( z \right)}}{{RT}}} \right) - 1} \right)} dz}}{{\int {_{{bound}}dz} }}} \right] - RT\ln \frac{{{V_{unbound}}}}{{{V^0}}} \approx - RT\ln \left( {{k_W}\exp \left( { - \frac{{\Delta W}}{{RT}}} \right)} \right) - {C_V}$$

where *z* is the reaction coordinate starting from zero and growing in a positive direction, *W(z)* is the function of potential energy dependent of reaction coordinate, Δ*W*—is an estimation of PMF computed accordingly to the Eq. (2) using a set of representative trajectories; because the summation for Δ*W* computations are done over the interesting (i.e. *W(z)* > > *0*) region of the reaction coordinate, the − 1 term in the middle statement of (3) is not essential. The integral in denominator defines the meaning of the complex bound state in terms of the one dimensional pulling process and its value is not important for the computation of the relative Δ*G* ^*o*^ for the series of homologous compounds as the meaning of their bound state is almost identical, but it is the major term for errors when comparing between chemically diverse structures. Although there are several theoretical studies about the nature of V_unbound_ and the integration limits in the PMF part [15, 40, 41], here we used a simpler empirical approach—we pre-computed PMF for three (benzimidazole) compounds with known affinity and fitted Eq. (3) against *k*_*w*_ and *C*_*v*_ constants. We also note that standard free energy of binding is typically of a little interest in actual drug optimization projects simply because it doesn’t change the predicted compounds potency ranking for derivatives within a series.

### Artificial neuronal network

The interpretation of molecular complexes is challenging problem because entropy, unlike potential energy, is difficult to quantify. We choose to develop a ANN to generalize the entropy contribution from observing the movement of the structure through MD. Thus, our approach for ANN architecture (Fig. 1) combines of two ideas: (i) the recognition of 3D structure, employing machine vision techniques, and (ii) classification of neural activity patterns in structure speculating layers with respect of potential energy produced by equilibrium molecular dynamic simulation at the speculated geometry.

The recognition sub-problem is solved with nested encode-decoder convolutional layers with two differences from the classic machine vision approach (i) molecular input images are 3D maps of voxels instead of 2D arrays of pixels and (ii) the ‘colors’ are used a generalized chemical properties (e.g. hydrophobic or hydrogen bonding potency) instead of reflecting photon wavelengths. In our protocol, the input voxel maps are constructed from atomic coordinates using an atomic density formalism ρ*(r)*:

4 $$\left\{ {\begin{array}{*{20}{c}} {r<{r_{ion}} \to \rho \left( r \right)=1,} \\ {{r_{ion}} \leq r<{r_{vdw}} \to \rho \left( r \right)=\exp \left( { - 2{{\left( {\frac{{r - {r_{ion}}}}{{{r_{vdw}} - {r_{ion}}}}} \right)}^2}} \right),} \\ {r>{r_{vdw}} \to \rho \left( r \right)=0.} \end{array}} \right.$$

where *r* is the distance from the center of the voxel to the given atom center, *r*_*ion*_ is ionic radius of the atom and *r*_*vdw*_ is its Van-der-Waals radius. The atomic density formalism ensures that the voxels within the ionic radii apart from the atomic center coordinates are set to the maximal absolute feature density value (equal to 1.0) and the other surrounding voxels are additively colored through a Gaussian curve centered at the atom coordinate (with a constraint that *ρ(r)* ≤ 1); the scale of the Gaussian curve area is set to 1.0 and its standard deviation to half of the difference between the ionic and Van-der-Waals radius of the atom. The densities maps from different atoms are additive but the absolute value of the sum is not allowed to exceed 1.0 per voxel. The atomic density is then projected onto one of the five different chemical features maps, depending of what the atom type is. The five input 3D maps are: a polar hydrogen density map (hydrogen bond donor map), hydrophobic motifs (carbon, chlorine, bromine and iodine atoms), nitrogen (hydrogen bond acceptor type 1), oxygen and fluorine maps (hydrogen bond acceptor type 2) and a d-elements map (sulfur and phosphor atoms). Finally, to improve the contrast of the molecular 3D images presented to the ANN, we mark ligand feature densities with a negative sign of feature density and receptor atoms with positive values; empty space is zero. In the ANN, the recognition process goes through three pairs plus one nested convolutional encoder-decoder layers (shown as boxes on Fig. 1 due to the 3D convolutional proximity). Because the goal of our ANN is to take into account the distribution of the complexes low-energy states (which is barely ‘visible’ from standalone 3D images), the classification was not computed for each 3D complex alone. Instead we trained the encoder-decoder component with a multitude of replications generated by parallel MD starting from the same docked pose; we also did not enforce any sparsity of feature maps [42] to capture more noise from emerging ‘misclassified’ patterns generated with training of multiple expected outputs per one input.

In order to account for entropy in ANN, we (i) trained an autoencoder using multiple structures after docking and after MD for each compound and (ii) connected a perceptron module (shown as flat vertical bars on Fig. 1) to the two innermost convolutional layers. The rationale of our design is that multiple structures per compound during training can be generalized by our deep encoder-decoder layers to represent the variety of states in the real system. The perceptron then classifies the emerging patterns which encode variety of states in the analyzed system and perceive the entropy. For this reason we avoid pooling layers in our CNN. ANN training was systematically repeated after new MD data was added to the training set. The conjugate gradient Polak-Ribiere optimization technique [43, 44] was used as a local minimizer and a Genetic algorithm as a global optimizer [45]. The combination is computationally intensive but, in our opinion, allows for the construction of a good classification which is free of ReLUs [46].

### Assessing PMF convergence

For assessing PMF convergence we monitored the evolution of free energy estimations as the simulation proceeded in time and when more statistics were collected. The approach considers umbrella sampling as a quasi-static process that continuously fixes errors introduced by a non-equilibrium pulling process. Our hypothesis is that after a short equilibration period, the parallel umbrella sampling fixes the errors uniformly and independently with respect to each other, so that the error fixing rate depends approximately only on the total amount of introduced errors. Our model of PMF convergence therefore considers a linear dependence of the amount of fixed errors at time *t* (measured as a finite difference ${{\Delta W\left( {t+\Delta t} \right) - \Delta W\left( t \right)}}/{{\Delta t}}$) and the convergence of the error fixing rate at time *t* (measured as the finite difference ${{\Delta \left( {\Delta W\left( {t+\Delta t} \right) - \Delta W\left( t \right)} \right)}}/{{\Delta {t^2}}}$) due to the decreased amount of all possible error that can be fixed. By replacing the finite differences with derivatives one can obtain the differential equation:

5 $$\frac{{{d^2}\Delta W\left( t \right)}}{{d{t^2}}}= - k\frac{{d\Delta W\left( t \right)}}{{dt}}$$

where the coefficient *k* stands for the linear regression parameter of the assessing PMF convergence model. The solution of Eq. (5) is a saturating (exponential decay) curve:

6 $$\Delta W\left( t \right)={C_0} - {C_1}\left( {1 - \exp \left( { - kt} \right)} \right)$$

for which parameters *C*_*0*_ and *C*_*1*_ are obtained by fitting to the empirical Δ*W(t)* table. Importantly, that function (6) has an asymptote $\mathop {\lim }\nolimits_{{t \to \infty }} \Delta W\left( t \right)={C_0} - {C_1}$ which theoretically allows extrapolation of PMF estimations to an infinite simulation time (when all errors are fixed) from reasonably long and accurate runs. Although the amount of available computational resources during Grand Challenge 2 was not sufficient for us to assess the PMF convergence extrapolation well, we were able to carry out good “sanity” checks for “unlucky” sets of routes encountered in the parallel umbrella sampling. As “unlucky” we consider a set of the four simulated routes that do not represent the lowest-most dissociation scenarios well. We believe that fixing of umbrella errors is a good place to observe the successful representation of low energy routes. Our method offers two additional kinds of the sanity checks (in addition to standard umbrella windows overlap check): (i) standard deviations in the set of parallel umbrella estimations (*σ*_*ΔW*_ in Eq. 2) and (ii) the value and sign of regression coefficient *k* in Eq. 6. As a marker of a good estimation we can use a small (but not too small) value of *σ*_*ΔW*_ together with a small positive value of *k*. The typical protocol is to compute PMF and estimations for entire series of compounds, gather statistics of *σ*_*ΔW*_ and *k* in the set and consider additional replications for compounds with the biggest (most suspicious) deviation.

### General logic of our protocols

Here we outline the overall logic of our multiple stages pose prediction and free energy estimation protocols.

**Fig. 2:**
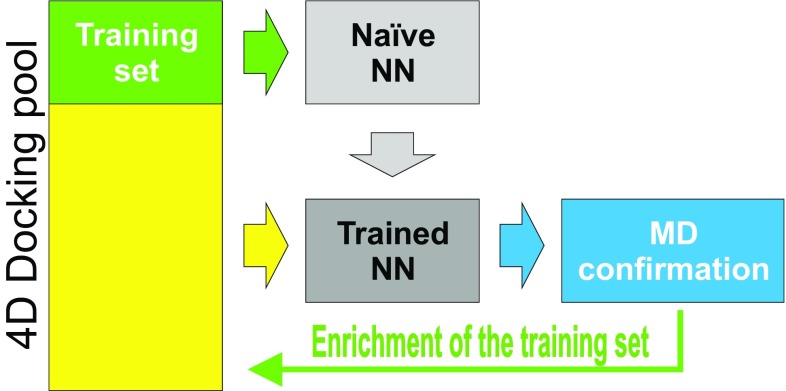
Artificial neuron network, 4D molecular docking and simulations of molecular dynamics are working together for the accurate prediction of FXR binding poses. A subset of complexes, used as a training set at the current iteration is shown in green; the yellow is the fraction of poses for which the evolution in time is predicted by the trained ANN learned from the training subset. The thin green arrow indicates the extension of the training set with new results from the MD simulation

Our iterative trainable pose prediction protocol (Fig. 2) combines energetic (in form of LIE) and geometrical similarity models. The initial training data set was composed of seven resolved bound compounds structures and five manually constructed complexes of their close homologues, with reported experimental activity. MD runs were computed (with eight replications) for each complex in the training set as it is described above. Complex geometries and interaction energies (i.e. <U^Coul^_Lig_>, <U^LJ^_Lig_>, <U^Coul^_Site_> and <U^LJ^_Site_>) were extracted for each compound and LIE model weight parameters were fitted against known experimental values. While interaction energies of the complexes were iteratively predicted by ANN, their corresponding LIE model weights were kept constant though the process. At each iteration, a naїve convolutional ANN (shown on Fig. 1) was trained to reproduce the voxels geometry of complexes after MD and the average interactional parameters of the LIE model using coordinates after docking as input. The trained ANN is then speculated voxels geometries and average interactional energies for every docked pose generated with 4D docking for 102 FXR compounds. The top binding poses per compound were selected from both geometrical and interaction channels of ANN. The energetic score was a sum of ANN predicted interaction averages weighted by constants from the LIE model and the geometrical score was similarity between the predicted voxels maps after MD and voxel maps constructed from coordinates of resolved complexes. The new top-scored pose candidates were analyzed with parallel MD runs. The new geometries after MD were converted into voxel maps and interactional averages were computed from the recorded trajectories. The simulation results for each of the newly analyzed poses were then used to enrich current training set (LIE weights were not recalculated). The iteration was then repeated till convergence, each time with a larger training set. After two iterations the algorithm converged i.e. the trained ANN could not predict any new top scored poses that were not simulated by MD during previous iterations. The best trajectories were eventually cooled down with simulated annealing to the temperature of liquid nitrogen and the final complex structures were sent to the D3R team for evaluation.

For the free energy protocol, we generated four dissociation trajectories per compound with non-equilibrium pulling at different temperatures and sampled each of them with quasi-equilibrium umbrella sampling. As the starting geometries in our pulling simulations were used poses predicted with our poses prediction protocol as it is described above. An important parameter of the pulling models—the direction vector of dissociation force—was set manually for one selected protein conformation and then all analyzed complexes were aligned on this selected structure with the manually defined dissociation vector. The sampled trajectories were analyzed with the WHAM method [47, 48] and by assessing the PMF convergence models we were also able to highlight the suspected outliers (although unfortunately we had no time to recalculate a better estimation for them).

## Results and discussion

**Fig. 3:**
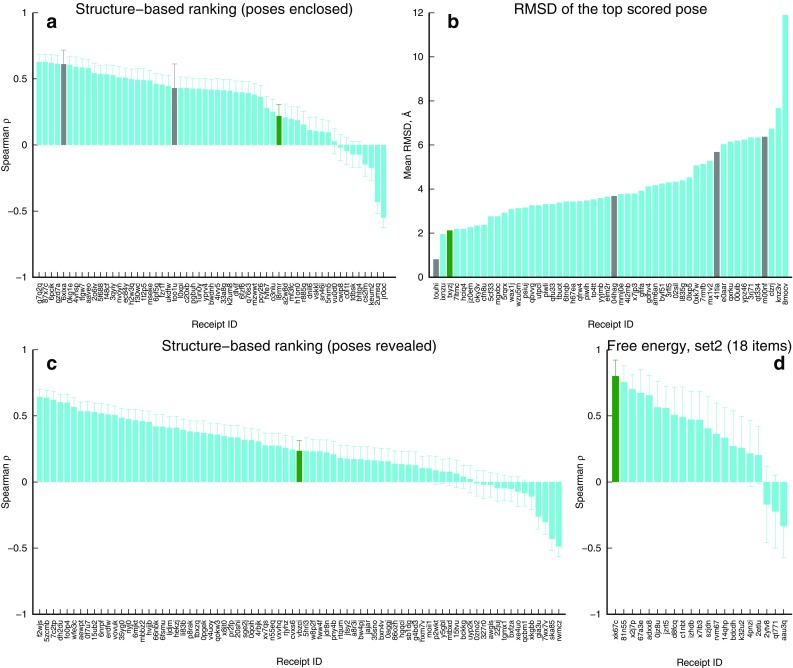
Performance of our screening techniques (green bars) in different sub-challenges. The results are sorted so the left most are the best and the grey bars refer to protocols with incomplete predictions. **a** Compounds scoring before releasing of the crystal structures, **b** binding pose predictions, **c** compounds scoring after the release of the crystal structures, **d** the evaluation of the free energy of binding

The Grand Challenge 2, the blinded drug modeling competition, held by the D3R online resource (https://drugdesigndata.org/) allowed us to evaluate several protocols in a consistent and unbiased way. We participated in four sub-challenges, all with a structure-based methodology: pose predictions, rapid compound ranking, rapid compounds ranking after using the correct binding pose and a computationally intensive (relative) free energy estimation. An important detail of the challenge is that experimental/literature data were available for only one of the three major chemotypes presented in the competition. Our results (summarized on Fig. 3) are particularly interesting for the pose prediction sub-challenge 3b and the free energy measurement sub-challenge 3d, where innovative protocols were applied, whilst compound scoring with classical LIE approach 3a and 3c was only of average accuracy.

Our approach for binding pose prediction was to combine machine learning with MD. Pose prediction is a necessary step for compound potency estimation when using a structure based approach. Therefore, we tested the approach with a simpler problem first before implementing a more complex scoring ANN. As we lacked a compound scoring machine learning protocol at the time of the competition, we used the LIE method for an estimation of compound binding potency. The method was chosen because it was also a component of the pose prediction pipeline. Although, the results of compounds scoring with LIE approach alone are not impressive, the method showed good accuracy as a component of the binding pose prediction pipeline. We therefore believe that our second place in the pose prediction sub-challenge is mostly attributed to the machine learning component which was able to generalize noisy LIE observations from multiple instances. Another important advantage of the ANN generalization is the ability to predict reasonable LIE parameters very quickly, spending less than a second per hundred of analyzed poses. This allowed us rescreen up to 60 poses per each compound on the fly and select the most promising poses in a manner similar to calculating multiple replica MD simulations in an explicit solvent. The prototyped method is of interest for high-throughput virtual screening because a typical compound database contains many millions of compounds, which can be analyzed quickly with an ANN, but contain a much smaller number of specific chemical classes which can be tractable to study using the much more computationally demanding MD studies.

**Fig. 4:**
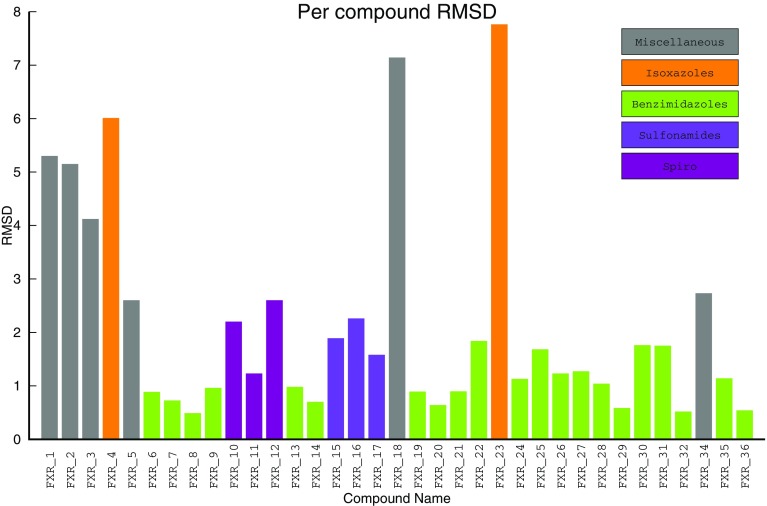
Accuracy of the pose prediction protocol (per-compound view). The four different chemotypes are shown in colors and stand-alone compounds are shown as grey

Our ANN showed good recognition of all the chemical structures, which were similar to those in the training set (Fig. 4). The initial (experimental) part of our training set was mostly composed of benzimidazoles, eight (3OKH, 3OKI, 3OOF, 3OOK and four more manually constructed) out of 13 structures, and all benzimidazoles (shown as green) are estimated with perfect average error 1.03 A without any dramatic outliers observed within a subset of 21 compounds. Conversely, other compounds (miscellaneous), where their chemotypes were not represented in the training set, displayed a much larger average error of 4.51 A with two good estimations apparently selected fortuitously. The non-trivial outcome within this challenge is that ANN provided good poses for spiro- derivatives (average error 2.01 A) and sulfonamides (average error 1.91 A). The errors are bigger than for benzimidazoles, but are much smaller than the errors for the miscellaneous compounds. Importantly, as neither of these two chemical classes (spiro and sulfonamides) had analogues in the training set, an ANN was dynamically trained to consider the outcome of MD simulations for the predicted binding of these classes. The reason why MD-based training did not work for miscellaneous compounds but worked for these two chemical classes, is likely because these two are represented as a small chemical series of three compounds each. This presumably allowed the ANN to generalize binding hypotheses, which were consistent for all members of the series. Another advantage of analyzing a series of derivatives is that should a promising binding pose be chosen (fortuitously) and successfully measured with MD for one of the members, the generalization of the ANN confers the estimation to every other series member. With the miscellaneous compounds, the generalization, if there were any, could not make useful predictions due to a lack of generalization of their individual spatial voxels patterns.

Isoxazoles represent another interesting example that illustrates vulnerability of our approach and which likely was detrimental to our performance in the pose sub-challenge. The two compounds (FXR_4 and FXR_23) form a series and are likely to be generalized together by ANN. Unfortunately, the two more isoxazoles series members from the initial training set (3RUT and one manually constructed) display significantly different binding modes. Guided by the similarity concept, our ANN classified new candidates to the existing wrong binding modes (average error is 6.89 A, even worse than for miscellaneous group) and no MD could compensate for such strong bias in the initial data. It would be interesting to ask whether an ANN could predict good poses for these two compounds from MD only, but, as it follows from the miscellaneous subset of compounds, it highly likely that the ANN would generate more accurate estimations if authors provided no misleading examples in the training data. It remains a large and unresolved problem as how to determine the criteria at which the experience gained from MD simulations can negate observations from a human-predefined initial training set.

In summary, there exists great potential for machine learning approaches to improve the present state of the art in the molecular modeling field. In our opinion, AI can supervise the rational design of compounds in a similar way to human medicinal chemistry experts but with the capacity to assess in detail many millions of compounds. Here we prototyped ANN for usage in high-throughput virtual screening systems. Our next goal toward implementing artificial intelligence for compound scoring is to elucidate the extremely difficult multiple-state and multiple-route entropy functions of binding to biological macromolecules where the AI is observing a large number of sampling events from the MD simulations of free energy estimations and applying generalizations in series in a similar manner as was used for the pose prediction challenge.

For compound ranking and for comparison with a non-ANN approach we used a classic LIE model. The LIE method is simple and relatively rapid compared to MD based scoring methods and possess rationale in form of maximizing the time-averaged (weighted) interactional energies. For entropy in short length MD runs this method involves regression parameters which are fitted from a representative training set. This also possesses probabilistic model of free energy estimations from a number of short runs to facilitate replicate sampling. The largest problem with LIE in this competition was the insufficient representation of binders in the training set. Only one chemotype (benzimidazoles, with minor inclusion of isoxazoles) out of the three dominating chemotypes from the set of ranked compounds had reported experimental data in the training set. This is likely to be responsible for the poorer performance of LIE—the relative predicted potency of the chemotypes showing no clear regression with experiment. Considering its rapidity and the good contribution to the pose prediction sub-challenge, we speculate that even an approximate fit of model parameters is sufficient to capture the maximum interactions trend in a series of chemical derivatives and thus LIE method can be of some use as a component of more complex MD-based affinity estimating protocols.

**Fig. 5:**
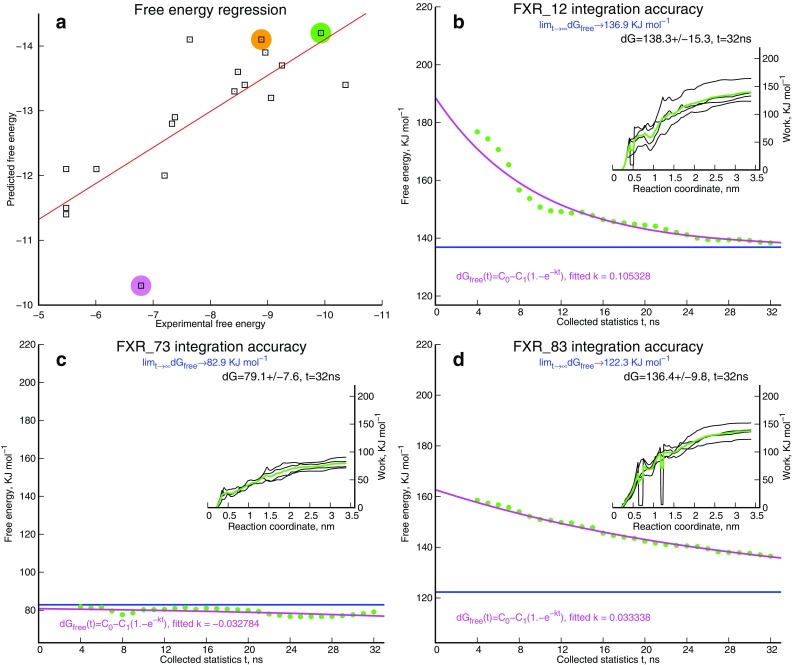
**a** Assessing PMF convergence helps to reveal difficulties in free energy estimations. **b** A well converged free energy estimation. **c** The largest expected underestimated outlier FXR_73 (violet circle), **d** the second largest overestimated outlier FXR_83 (orange circle) shows significant deviations from the well-estimated FXR_12 (green cycle)

For the free energy sub-challenge, we used own technique that combines multiple non-equilibrium pulling simulations and the subsequent studying of the trajectories with quasi-equilibrium umbrella sampling. The approach is computationally intensive but the estimations are competitive (Figs. 3d, 5a). The main idea of our protocol is to account for the probabilistic nature of each estimation. Considering the multitude of association (dissociation) trajectories, there is neither a correct nor an impossible search scenario. Different independent estimations of free energy from the MD simulations are all perfectly valid (within accepted modeling approximations). The observed potency (free energy) of binding comes from weighted averaging over an ensemble of possible dissociation scenarios. The logic of our approach is to generate several different scenarios—limited only by the available computational power—and study them all together to determine what the weighted average should it be. This is done by generating distinct dissociation trajectories with multiple pulling experiments and then smoothing the entire set with a quasi-equilibrium sampling protocol. Due to prohibitive computational cost, we considered a smaller amount of routes and quasi-equilibrium smoothing runs than is required to saturate the accuracy of the theoretical estimations. For example, we generated only four independent dissociation trajectories for sampling instead of a more desirable 16 per compound and sampled each trajectory for 32 ns instead of approximately 50 ns. Therefore, we believe that even better accuracy for our free energy method could be achieved giving a larger investment in computation.

Because of limited computational resources and for efficiency in lead optimization, one can not expect an (umbrella) sampling protocol that achieves accuracy to be totally independent from the starting conditions provided by non-equilibrium pulling process. This means contamination of the estimations with errors caused by additional energy which is silently borrowed from external thermostat. To visualize the borrowed energy contribution we propose the use of replicate umbrella samplings for a representative set of low-energy dissociation scenarios. Our idea is that each estimation consists of a constant free energy term and variable amount of energy borrowed from the external bath. The amount of borrowed energy is mostly dependent on the dissociation scenario so averaging over several sampled scenarios should compensate for the error. In addition, averaging along the lowest energy routes is expected to be the most accurate because passing of any additional energy hills along a dissociation route decreases sampling windows overlap in generalized orthogonal coordinates. We therefore try to sample the most probable subset of routes with the expectation that such estimations are the most accurate, but, as this is not guaranteed, error tracking is necessary. While errors in the state sampling are monitored by standard umbrella, the route sampling errors are more difficult to detect. To reveal errors caused by insufficient sampling in dissociation routes we assessed PMF convergence and detected poorly converging averages by observing their convergence history. By combining these two techniques we ensure that (i) the limited multiple replica approach provides a reasonably accurate estimation of binding affinity for many compounds with a minimal amount of required computation and (ii) assessing PMF convergence reveals incorrectly estimated outliers that will require additional study. Because the majority of the estimation errors are contributed by the “unlucky” selection of subsets of pulling scenarios (we assume that convergence in overlapping of umbrella windows is successfully confirmed), one can monitor the time-dependent convergence of free energy estimations in order to measure the uniformity of error correction in the quasi-equilibrium phase of the analysis. If the routes of sampling are balanced, then the errors are uniformly fixed so that the average estimation of parallel umbrella sampling smoothly converges in accordance with an exponential decay law (e.g. the case of FXR_12 compound, Fig. 5b). Otherwise the convergence rate becomes abnormally slow (as in case of FXR_83, Fig. 5d) or does not follow an expected behavior (as in FXR_73, Fig. 5c); either of which indicates poor accuracy of the resultant estimations. Although we did not know the correct free energy values at the time of submission, we added these three time-dependent diagrams to our protocol to illustrate our knowledge about the largest incorrect estimations within the blind test. Ultimately, FXR_73 was our most discrepant outlier, FXR_83 is the second largest overestimated outlier (although we had no evidence that FXR_81 was incorrectly over-estimated) and FXR_12 was an accurately estimated case. In a real drug design project, we would consider repeating the computations for the suspected outliers with new starting seeds which (eventually) should provide more balanced routes sampling and more accurate predictions.

## Conclusions

Here we have demonstrated two promising approaches to improve quality of computer-aided drug design: (i) applying of ideas from the machine vision field to incorporate knowledge about molecular complex behavior from MD simulations into high-throughput virtual screening and (ii) improve the free energy sampling quality of the umbrella method with multiple non-equilibrium dissociation trajectories and assessing PMF convergence for detecting outliers. During Grand Challenge 2 we also determined areas, especially in high-throughput compound scoring and ranking, where our technological approaches are now being improved.
